# The Human Behaviour-Change Project: An artificial intelligence system to answer questions about changing behaviour

**DOI:** 10.12688/wellcomeopenres.15900.1

**Published:** 2020-06-10

**Authors:** Susan Michie, James Thomas, Pol Mac Aonghusa, Robert West, Marie Johnston, Michael P. Kelly, John Shawe-Taylor, Janna Hastings, Francesca Bonin, Alison O’Mara-Eves

**Affiliations:** 1 Centre for Behaviour Change, University College London, London, UK; 2 UCL Institute of Education, University College London, London, UK; 3 IBM Research, Dublin, Ireland; 4 Research Department of Epidemiology & Public Health, University College London, London, UK; 5 Aberdeen Health Psychology Group, University of Aberdeen, Aberdeen, UK; 6 Primary Care Unit, Institute of Public Health, University of Cambridge, Cambridge, UK; 7 Centre for Computational Statistics and Machine Learning, University College London, London, UK

**Keywords:** behaviour change, artificial intelligence, ontologies, interventions, evidence synthesis

## Abstract

Changing behaviour is necessary to address many of the threats facing human populations.  However, identifying behaviour change interventions likely to be effective in particular contexts as a basis for improving them presents a major challenge. The Human Behaviour-Change Project harnesses the power of artificial intelligence and behavioural science to organise global evidence about behaviour change to predict outcomes in common and unknown behaviour change scenarios.

Changing behaviour is necessary to address many of the threats facing human populations. However, identifying behaviour change interventions likely to be effective in particular contexts as a basis for improving them presents a major challenge. The Human Behaviour-Change Project (HBCP) aims to support decisions about interventions using cutting-edge ***artificial intelligence*** (AI) (please see Glossary, Table 1) to collate, synthesise and interpret evidence from behaviour change intervention evaluations. This Wellcome Open Research Collection editorial introduces the HBCP, explains the rationale for the project, summarises the contribution of the core areas of expertise (behavioural science, computer science and system architecture), and outlines what it aims to deliver.

**Table 1.** Glossary.

Term	Definition	Source
**Artificial Intelligence**	The theory and practice of building computer programs to perform tasks that a human would reasonably regard as requiring intelligence.	Nilsson (2014)
**Knowledge System**	An automated system delivering comprehensive, high quality, timely and accessible syntheses and interpretations of evidence in the domain of behaviour change.	Michie *et al.* (2017)
**Machine Learning**	Computer algorithms that learn from sample inputs and apply that learning to make predictions on data or classify data into categories.	Alpaydin (2014)
**Natural Language** **Processing**	Algorithms that extract meaning from passages of text in a form that can be used for inference by computers.	Chowdhury (2003)
**Ontology**	A standardised representational framework providing a set of terms for the consistent description (or “annotation” or “tagging”) of data and information across disciplinary and research community boundaries.	Arp *et al.* (2015)

## The problem: understanding and improving behaviour change interventions

Policy makers, planners and practitioners seek answers to complex questions when it comes to behaviour change interventions; these are typically variants of what may be termed the ‘big question’: What works, compared with what, for what behaviours, how well, for how long, with whom, in what setting, and why?’ ( Michie *et al.*, 2017). Until now, the main scientific method for addressing such questions has involved conducting systematic literature reviews and synthesising findings by meta-analyses where possible.

While this approach has proved hugely valuable, it has a number of limitations. First, intervention evaluations are being published on a vast and accelerating scale, which is beyond human capability and resources to analyse with the breadth, granularity and timeliness required. Second, intervention effects may be different in different populations and settings, so analyses need to address potentially complex interactions. Third, variations in study methodology and reporting practices make comparison across studies problematic. The final point reflects the lack of a shared conceptual and linguistic framework in the field. The HBCP seeks to address these limitations.

## The Human Behaviour-Change Project

The Human Behaviour-Change Project is a collaboration of behavioural and computer scientists and system architects that will create an *AI-based **Knowledge System*** capable of scanning the world’s published reports of behavioural intervention evaluations, extracting and analysing relevant information organised by a Behaviour Change Intervention Ontology (BCIO) and answering specific user queries, indicating the level of confidence in the answers and explaining the process. The first behaviour we are investigating is smoking cessation, drawing on published reports of randomised controlled trials.

The key activities involved in creating the HBCP Knowledge System involve developing:

1. An ***ontology*** (see Glossary, Table 1) of behaviour change interventions and evaluation reports.
2. A largely automated system to identify relevant research as it is published, and extract entities appearing the BCIO from behaviour change intervention evaluation reports using ***natural language processing*** (see Glossary, Table 1).
3. A representation of knowledge in evaluation reports structured according to the ontology.
4. Reasoning and ***machine learning*** (see Glossary, Table 1) algorithms to synthesise this information and make inferences in response to user queries.
5. An interface for computers and human users to interact with the system.

For further information, see https://www.humanbehaviourchange.org/, Michie *et al.* (2017), and Norris *et al.* (2018).

## The contributions of behavioural science, computer science and system architecture

### Behavioural science

The Behaviour Change Intervention Ontology (BCIO): from printed to computer-readable research reports

The behavioural science component of the project aims to make published reports of interventions not only readable by humans but readable by computers. To be computer readable, intervention reports need to be translated into a standardised, scientific language to overcome the heterogeneity in the way reports are currently written. The HBCP needs to represent the knowledge contained in these reports as formal entities and relationships, i.e. as ‘ontologies’, that can work with other, related knowledge representations. The HBCP ‘Behaviour Change Intervention Ontology (BCIO)’ is being developed to characterise interventions, their contexts and evaluations and currently includes more than 2000 entities. This is an exercise which seeks to ensure interoperability with other ontologies such as the Cochrane PICO ontology and that as far as possible existing terms are used rather than new ones being created.

The BCIO combines the knowledge of behavioural and social science experts about evidence, theory and practical experience of behaviour change interventions and evaluations with scrutiny of published reports of behaviour change intervention evaluations. For each BCIO ontology, more than 400 reports were annotated for about 100 entities in a computer-readable form by behavioural experts, prioritising entities that were frequent in reports and likely to be useful in training the knowledge system (see Table 1 and next section). Ontology and behavioural science experts provided feedback leading to an iterative process of further annotation, expert feedback and redrafts by the behavioural science team.

Accompanying papers describing the development of the structure, or upper level, of the BCIO along with papers on ontologies for mode of delivery, setting, and population are presented in the HBCP Collection.

### Computer science: from research reports to prediction

The computer science component of the project aims to extract, synthesise and make predictions from computer readable research reports using machine learning. The original intention was to automatically extract data corresponding to entities in the BCIO from intervention evaluation reports with near 100% accuracy using natural language processing, machine learning and statistical reasoning. However, the quality and variability of evaluation reports and the diversity of language used together with the number of data items to be extracted presented a significant problem. Initial progress was promising but achieving near 100% accuracy would have required an unrealistic amount of resources and time as well as evidence from many more studies (assuming that sufficient studies about each intervention have actually been conducted).

We therefore developed a novel approach to evidence synthesis, making predictions about hypothetical scenarios from large volumes of less accurate annotations. This approach, termed Prediction Algorithms from Vector Space (PAVeS), extracts less than perfect annotations and represents each BCIO scenario as an entity in computable format in a high-dimensional vector space.

By applying similarity estimation techniques over this vector space, hypothetical outcomes together with associated confidence scores are identified, allowing comparison of interventions and the selection of those likely to achieve a better success rate in a certain situation with an associated confidence score.

In PAVeS, analytical exactness is traded off for improved flexibility and scalability. For example, gaps are permitted in PAVeS when the computer cannot extract some values from a report, so reaching a “good enough” representation which will be reflected in the confidence scores. The PAVeS approach exploits the computational capability of machine learning (ML) to process data at massive scale, while continuously incorporating new material into its evidence base (see system architecture). The BCIO provides an *a priori* knowledge framework which reduces computational demands on the ML algorithms by limiting what entities may be associated with each other and by setting starting conditions for certain ML parameters.

### System architecture and the user interface: from data to decisions

The system architecture component of the project connects the international research literature with the BCIO and PAVeS and connects the users with the knowledge output from PAVeS. There are two discrete components of the work involved. First, we need to ensure that the PAVeS-based system is able to draw on a comprehensive and up to date set of research data. In order to do this, we have developed a system that automatically pulls in as much of the world’s research from open sources as possible. This activity results in a dataset of several hundred million research records. The much smaller set of records relevant to the HBCP is selected for input to PAVeS using machine learning. Secondly, a user interface is being built to enable diverse users to interact with the current world of evidence contained in this dataset, and the predictions derived from it, in order to make choices amongst possible behaviour change interventions, and reach decisions based on valid estimates of confidence of their likely success in the user’s context. This component of the project is necessarily the last to be developed, given that it requires the availability of the other components in order to operate.

## Evaluating the HBCP

We will evaluate the HBCP Knowledge System on a number of dimensions, including: performance outcomes of the system (e.g. accuracy of information extraction), cost-effectiveness, ease of use and clarity of the system, and the credibility and trustworthiness of the system outputs. Having first identified likely end-users and interested groups, we have developed ‘personas’ to determine the likely information needs and user experience requirements of each. A series of evaluation protocols have been developed that consider the personas, the data to be collected, and the timing of the data collection, in order to best inform the project development and aid with dissemination and uptake of the system.

## What the HBCP will deliver

The HBCP aims to deliver a live system for open use to provide knowledge about (i) variations in behaviour change interventions and factors affecting their success in achieving intended outcomes, and (ii) how such a system might be taken forward in the future.

The HBCP will deliver an end-to-end system including a full life-cycle to

- identify relevant world literature
- represent BCIO knowledge in ontologies that link to other ontologies
- extract entities appearing in the BCIO from study reports via natural language processing
- train AI entity-extraction algorithms and learning via initial human annotation of literature
- analyse the information flexibly and at scale using PAVeS to produce knowledge of likely effectiveness of behaviour change interventions
- provide an interface for human and machine users to pose questions and receive possible answers with confidence estimates i.e. a machine generated judgement of the probability that predictions are correct and explanations of how the conclusion was reached, and
- integrate end-user feedback to further train the knowledge system.

The first phase of the HBCP will achieve a proof of principle in one behavioural domain, smoking cessation, and will explore the application of the knowledge system to a very different set of behaviours: those required to increase physical activity.

## Conclusions

As a result of addressing the practical challenges encountered in designing and delivering the Knowledge System, the HBCP is contributing to knowledge in several fields. The Behaviour Change Intervention Ontology enables such a wide and disparate field as the evaluation of behaviour change interventions to have a shared structure and language. This enables clarity of thinking, full reporting, evidence synthesis and linkage to other sciences.

The project aims to evaluate whether it is possible to make good predictions of variables of interest on unseen studies, based on partial and potentially imperfect information that can be extracted from intervention reports. This has been a resource intensive activity in terms of data resources and the development of technical solutions. While there are challenges and costs involved in adopting the BCIO and using it to extract information from world literature, the alternative of continuing with current approaches to a heterogeneous, disparate literature requiring human synthesis is also costly, wasteful, and does not build cumulative knowledge.

Current machine learning technologies cannot mimic humans in this problem space, but potentially do not need to. Our work in designing the system to support this project has developed solutions that advance the field of data science and its use of large-scale but ‘noisy’ bibliographic datasets, in order to deliver a platform that is able to keep up with the pace of research publication. Innovative machine learning and reasoning algorithms will make much better use of available data to make predictions about outcomes of behaviour change interventions. As the work proceeds and as further iterations with other forms of behaviour are developed, we anticipate improvements in the efficiency and accuracy of predictions. We also anticipate advances in system architecture, for example, in identifying behaviour change interventions from the world literature with greater accuracy. This will lead to key knowledge resources to inform policy and practice.

In summary, the HBCP will deliver a usable knowledge and prediction system for smoking interventions, illustrate the wider potential of such systems, clarify what additional science is needed to advance in each of the relevant fields, contrast small precise with large imprecise data as part of future science evidence, and consolidate the role of ontologies as a tool to advance behavioural science, promote scientific collaboration as well as to provide practical solutions. We hope that this Collection of papers will provide accessible information for their understanding and use.

## Data availability

### Underlying data

No data are associated with this article
